# The relationship between digital literacy and college students’ academic achievement: the chain mediating role of learning adaptation and online self-regulated learning

**DOI:** 10.3389/fpsyg.2025.1590649

**Published:** 2025-07-03

**Authors:** Fanfeng Chen

**Affiliations:** School of Mathematics, Hangzhou Normal University, Hangzhou, China

**Keywords:** academic achievement, digital literacy, learning adaptation, online self-regulated learning, higher education

## Abstract

**Object:**

This study explores the relationship between digital literacy and college students’ academic achievement and focuses on the mechanism of learning adaptation and online self-regulated learning.

**Method:**

A total of 894 college students were tested using digital literacy, learning adaptation, self-regulated learning, and academic achievement scales. SPSS 26.0 and Amos 24.0, structural equation modelling were used to examine the direct and interactive effects of these variables on academic achievement.

**Results:**

The findings demonstrated that digital literacy significantly and positively predicted college students’ academic achievement. Furthermore, both learning adaptation and online self-regulated learning were found to individually mediate this relationship. Notably, a sequential chain mediation was also confirmed: digital literacy enhanced learning adaptation, which subsequently fostered online self-regulated learning, ultimately leading to improved academic achievement. The results suggest the important factors affecting the academic achievement of college students, help improve their academic achievement, and provide useful suggestions for the education and teaching strategies of higher education management departments and educators.

## 1 Introduction

College students’ academic achievement is regarded as an important indicator of success for individuals, schools, universities, and even nations (Nyström et al., 2019). Academic achievement is usually measured by students’ scores and GPA (York et al., 2015), but also encompasses broader competencies such as students’ critical thinking skills, problem-solving skills and the ability to apply learned knowledge to practical situations (Cole, 1990). Studies have shown that factors such as appropriate digital engagement, personal differences, and interaction with the learning environment can affect their academic functioning (Hietajärvi et al., 2022). In the current wave of information technology in the 21st century, the importance of data has become increasingly prominent. Consequently, the relationship between data, information, knowledge, and wisdom, along with the impact of these changes on individuals, organizations, and society, becomes a focus of discussion (Atkinson et al., 2013). Digital literacy is recognized as a key component of a 21st century competency framework (Martínez-Bravo et al., 2022). The United Nations Educational, Scientific and Cultural Organization is committed to improving digital literacy worldwide through projects and initiatives, such as leveraging information and communication technologies to promote equity in education (Lyu and Yao, 2021). The application of digital technology in education has become a significant new development direction (Sousa et al., 2020), as the progress of technology has effectively promoted the dissemination and sharing of knowledge, and the channels of knowledge dissemination have become unprecedentedly diversified and convenient.

However, the application of digital technology in education also carries certain risks. College students need to acquire the necessary digital skills to adapt to new learning methods. In this context, digital literacy extends beyond the mere ability to work with computers; it also includes the capacity to process large amounts of information, assess its reliability, and critically examine digital content and environment, recognizing potentially embedded perspectives (Eshet-Alkalai and Soffer, 2012).

Digital literacy is regarded as a key element of contemporary education strategy (Erstad et al., 2021). Studies have shown that academic achievement is strongly correlated with students’ adaptation to college life (Iliichuk and Vorobets, 2020). Furthermore, from a motivational standpoint, Self-Determination Theory (Deci and Ryan, 2012) offers a valuable lens, positing that fulfilling students’ basic psychological needs for autonomy, competence, and relatedness can foster intrinsic motivation and promote more engaged and self-regulated behaviors, which are crucial for academic success. High digital literacy can promote essential student competencies, such as independent knowledge acquisition, critical thinking and teamwork (Tekesbaeva et al., 2023), potentially by enhancing their sense of competence and autonomy in the learning process. This capability greatly facilitates their adaptation to the digital learning environment. Consistent with this, research indicates that self-regulated learning, a behavior often linked to feelings of autonomy and competence, is a positive predictor of academic achievement (Cazan, 2014). In summary, existing studies have shown that digital literacy will directly or indirectly affects students’ academic achievement. However, there is a need of further research on the specific indirect pathways through which digital literacy influences academic achievement, particularly considering potential motivational mediators like adaptation and self-regulation.

The main purpose of the present study is therefore to explore the relationship between digital literacy, learning adaptation, online self-regulated learning and academic achievement. The research focuses on the following questions: (1) Do digital literacy, learning adaptation and online self-regulated learning have significant effects on academic achievement? (2) How does digital literacy affect academic achievement through learning adaptation and online self-regulated learning? The answers to these questions aims to provide valuable insights for educational practice and policy formulation.

## 2 Literature review

Digital literacy, introduced by Paul Gilster, refers to the ability to understand and use information in various forms from diverse sources accessed via computers and other digital devices (Gilster, 1997). Admist the rapid development of digital technology, digital literacy has become an indispensable core competency for students in the modern education system. Digital literacy is a comprehensive ability that extends beyond mere technical operation, encompassing an understanding of the digital environment, critical thinking, ethics considerations, and other cognitive and socio-emotional aspects (Erstad et al., 2021). For college students, digital literacy entails the ability to effectively identify information, solve problems, think creatively and learn new skills amidst vast network information resources (Park et al., 2021). This competency is crucial for individuals to adapt to the rapidly changing digital and technological environment and effectively use digital resources to enhance personal learning and career development.

Current research highlights not only the operational aspects of using digital technologies but also emphasizes students’ abilities to effectively acquire, evaluate, create, and communicate information in complex information environments (Eshet, 2004). This broad definition positions digital literacy as an important variable influencing academic achievement, as it directly relates to students’ capacity to effectively utilize digital resources for learning and knowledge application (Gilster, 1997). Furthermore, research indicates a significant positive correlation between digital literacy and academic achievement. Specifically, research by Pangrazio and Selwyn (2019) reveals that high digital literacy enhances students’ information filtering and analysis skills, thereby improving their academic performance. In addition, digital literacy positively influences students’ self-efficacy, autonomous learning, and learning motivation, which in turn promotes academic success (Tondeur et al., 2016). However, while existing literature indicates that digital literacy has a significant positive impact on academic achievement, the specific mechanisms underlying this relationship warrant further exploration and validation. Therefore, this study proposes the following hypotheses:

Hypothesis 1 (*H1*): Digital Literacy can significantly and positively predict college students’ academic achievement.

Learning adaptability is increasingly recognized as a key mediating variable in the relationship between digital literacy and academic achievement. Learning adaptability reflects students’ capacity to quickly adjust their learning strategies and behaviors in response to the constantly changing learning environments (Martin et al., 2013). This adaptation process is influenced by various factors, including the educational environment, learning motivation, perception of academic well-being, and professional curriculum characteristics (Corti et al., 2023). With the widespread adoption of digital learning tools, the importance of learning adaptability has become increasingly evident. Boekaerts (1999) emphasized the necessity of adaptive learning in the digital age, highlighting hat students need to continuously adapt to new technologies and learning methods to achieve academic success. Previous studies have shown a close relationship between digital literacy and learning adaptability. Students with high digital literacy tend to adapt more effectively to changing learning environment and use digital tools flexibly; these capabilities can positively influence academic performance fostered by digital literacy (Chaw and Tang, 2024). In addition, Vermunt and Verloop (1999) confirmed that students with high learning adaptability can effectively adjust their learning strategies when facing academic challenges, maintain a high level of academic engagement, and consequently achieve better academic performance. Nevertheless, the mediating role of learning adaptability in the link between digital literacy and academic achievement has not been thoroughly explored. Therefore, this study explores the mediating role of learning adaptability between digital literacy and academic achievement.

Hypothesis 2 (*H2*): Learning adaptation mediates the relationship between digital literacy and academic achievement.

Beyond adaptability, in the digital education environment, online self-regulated learning (OSRL) has emerged as a prominent area of academic research. Online self-regulated learning involves the learner’s capacity to actively and constructively manage, guide, and control complex learning activities (Kauffman, 2004). With the rapid development of online learning resources and platforms, students’ ability to effectively self-regulate in digital environments has become a key factor influencing academic achievement (Azevedo and Cromley, 2004). Online self-regulated learning encompasses not only setting learning goals and selecting appropriate learning strategies but also monitoring learning progress and reflecting on and adjusting learning methods (Zimmerman, 2002). Multiple studies have shown that students with higher digital literacy generally exhibit greater online self-regulated learning skills. This enhanced self-regulation enables them to use online resources and tools more effectively, schedule their learning autonomously, and be more self-aware, thereby improving learning outcomes (Broadbent and Poon, 2015). Dabbagh and Kitsantas (2012) emphasized the importance of online self-regulated learning in enhancing students’ learning flexibility and their ability to cope with learning challenges. Furthermore, Artino (2008) pointed out that students with strong self-regulated learning abilities in online environments are often better at controlling their learning environment and managing their time, thereby maintaining clarity in their learning motivation and goals. These studies suggest that online self-regulated learning may play an important mediating role in the relationship between digital literacy and academic achievement. However, this hypothesis still requires further validation.

Hypothesis 3 (*H3*): Online self-regulated learning mediate the relationship between digital literacy and academic achievement.

Learning adaptability and online self-regulated learning are two important variables influencing academic achievement that have garnered significant attention. Previous research suggest that each may individually mediate the relationship between digital literacy and academic achievement. This study further explores whether learning adaptability and online self-regulated learning might operate sequentially in a chain mediation model, linking digital literacy to academic achievement. Specifically, it is proposed that students with high learning adaptability are better positioned to master digital tools and adapt to new learning environments, which in turn enhances their online self-regulated learning abilities (Dweck and Leggett, 1988). This enhanced self-regulated learning ability further subsequently assist students in effectively managing their learning pace and adjusting strategies in digital learning environments, thereby improving academic achievement (Pintrich and De Groot, 1990). Moreover, the interconnectedness of adaptability, self-regulation, and academic success in diverse learning contexts is well-documented, underscoring the importance of these student attributes (Hadwin et al., 2011). Based on theoretical rationale, this study proposes the following hypotheses.

Hypothesis 4 (*H4*): Learning adaptability and online self-regulated learning sequentially mediate the relationship between digital literacy and academic achievement, such that digital literacy enhances learning adaptability, which in turn improves online self-regulated learning, ultimately leading to higher academic achievement.

## 3 Methods

### 3.1 Participants and procedure

A total of 1, 207 questionnaires were distributed to university students in Zhejiang Province, China, using a convenience sampling method. After excluding invalid responses (e.g., incomplete questionnaires), 894 valid questionnaires were retained for analysis, yielding a response rate of 74.06%. The final sample consisted of 466 male participants and 428 female participants. In terms of academic year, the sample included 369 freshmen (41.3%), 301 sophomores (33.7%), 126 juniors (14%), and 98 students who were seniors or in higher years (11.0%).

Prior to participation, students were informed that their involvement was voluntary and that all responses would remain anonymous. Written informed consent was obtained from all participants. The study procedures involving human participants received ethical approval from the Academic Committee of Hangzhou Normal University.

### 3.2 Measurements

#### 3.2.1 Digital literacy scale

Digital literacy was assessed using a scale adapted for the Chinese context. While drawing on established international frameworks (Eshet, 2004), the instrument was significantly informed by local research (Sheng, 2022) to enhance cultural relevance. Scale items were underwent reviewed for cultural suitability and linguistic clarity, followed by a pilot testing with a small sample of Chinese university students to confirm item comprehensibility prior to the main study. The final digital literacy scale in this study comprised 13 items across four dimensions. The information domain (3 items, e.g., “I can understand the basics and methods of information retrieval and accurately obtain the required information”), the communication domain (3 items, e.g., “I can share data, information, and digital content with others through appropriate digital technologies”), the content creation domain (3 items, e.g., “I can use digital tools to create and edit various digital content to express my ideas”), and the problem-solving domain (4 items, e.g., “I can identify technical issues when operating devices and using digital environments, and solve these issues”). Participants rated items on a 5-point Likert scale, ranging from 1 (Strongly Disagree) to 5 (Strongly Agree). Higher total score indicates greater digital literacy. In the present study, the Cronbach’s alpha coefficient for this scale was 0.96.

#### 3.2.2 Learning adaptation scale

Learning adaptability was measured using College Student Learning Adaptation Scale compiled by Feng et al. (2006). The scale contains 18 items, primarily covering five dimensions: learning motivation (4 items, e.g., “I feel that I adapt to university learning”), teaching mode (4 items, such as “The teaching methods of university instructors often make me feel uncomfortable”), learning ability (3 items, e.g., “After entering university, my way of thinking has become more mature”), learning attitude (3 items, e.g., “University learning relies solely on personal interest, and no specific methods are needed”), and learning environment (4 items, e.g., “The living conditions in university have a significant impact on learning”). Responses were provided on a 5-point Likert scale, ranging from 1 (Strongly Disagree) to 5 (Strongly Agree). After appropriate reverse-scoring, higher total scores indicate greater learning adaptability. In the present study, the Cronbach’s alpha for this scale was 0.91, demonstrating high internal consistency.

#### 3.2.3 Online self-regulated learning scale

The Online Self-Regulated Learning Scale, originally developed by Lynch and Dembo (2004) and Barnard et al. (2009), underwent a specific localization process for this study. This involved a rigorous translation and back-translation procedure, followed by an expert review panel consisting of educational psychologists and instructional technologists familiar with the Chinese higher education online learning landscape. Five dimensions include goal setting (4 items, e.g., “I set goals to help me manage my online learning time”), environment construction (3 items, e.g., “I clearly know where I can conduct the most effective online learning”), task strategies (4 items, e.g., “before entering forums or chat rooms for discussion and communication, I will prepare my questions”), time management (3 items, e.g., “I try to plan my daily or weekly study time and follow this plan for online learning”), and seeking help (3 items, e.g., “when I need assistance, I consult classmates who are more familiar with the course content”). The scale consists of 17 items and employs a Likert 5-point scale from 1 (Strongly Disagree) to 5 (Strongly Agree). A higher score indicates a stronger ability of college students in self-regulated online learning. In this study, the Cronbach’s alpha coefficient of the scale was 0.93.

#### 3.2.4 Academic achievement scale

This study utilized the College Student Academic Achievement Scale developed by Zhou (2020). The scale mainly includes three dimensions: learning performance (3 items, e.g., “I can complete learning tasks within the specified time”), academic contribution (3 items, e.g., “I actively seek challenging learning tasks”), and interpersonal facilitation (3 items, e.g., “I actively participate in team collaboration”), totaling 9 items. A Likert 5-point scale was used from 1 (Strongly Disagree) to 5 (Strongly Agree). A higher score indicates a higher academic achievement among college students. The Cronbach’s alpha coefficient of this scale was 0.902.

### 3.3 Statistical analyses

Structural equation modelling (SEM) using AMOS 24.0 was selected as the primary analytical technique for this study. SEM allows for the simultaneous examination of: (1) the direct effect of digital literacy on academic achievement; (2) the simple mediating effects of learning adaptation and online self-regulated learning individually; and (3) the chain mediating effect where digital literacy influences academic achievement sequentially through learning adaptation and then online self-regulated learning. This approach is superior to separate regression analyses as it accounts for measurement error in the latent constructs (represented by the scale items), tests all hypothesized paths within a single, integrated model, and provides overall model fit indices to assess the plausibility of the proposed theoretical framework. The Bootstrap procedure (with 5,000 samples) was further employed within SEM to robustly test the significance of the specific indirect (mediating) effects.

## 4 Results

### 4.1 Testing for common method bias

To assess potential common method bias (CMB), Harman’s single-factor test was conducted (Zhou and Long, 2004). An exploratory factor analysis (EFA), including all items from the study’s questionnaires, was performed. According to this technique, CMB may be a concern if a single factor emerges from the analysis or if one general factor accounts for the majority of the covariance among measures. The results show that there are 9 common factors with eigenvalues greater than 1, among which the maximum factor explains a variance of 30.45% (less than 40%). In summary, this study does not exhibit significant common method bias.

### 4.2 Descriptive statistics and correlation analysis of each variable

Table 1 presents the means, standard deviations, and Pearson correlation coefficients for the key study variables: digital literacy, learning adaptation, online self-regulated learning, and academic achievement. As shown in the table, all variables were significantly and positively intercorrelated (all *p* < 0.01). Specifically, digital literacy demonstrated significant positive correlations with learning adaptation (*r* = 0.487, *p* < 0.01), online self-regulated learning (*r* = 0.526, *p* < 0.01), and academic achievement (*r* = 0.526, *p* < 0.01). Furthermore, learning adaptability was positively correlated with both online self-regulated learning (*r* = 0.624, *p* < 0.01) and academic achievement (*r* = 0.680, *p* < 0.01), and online self-regulated learning showed a strong positive correlation with academic achievement (*r* = 0.712, *p* < 0.01).

**Table 1:** Correlation analysis results among the variables (*n* = 894).

Variable	*M*	SD	1	2	3	4
1. Digital Literacy	3.56	0.66	—			
2. Learning adaptation	3.43	0.44	0.487**	—		
3. Online self-regulated learning	3.23	0.58	0.526**	0.624**	—	
4. Academic achievement	3.48	0.58	0.526**	0.680**	0.712**	—

*p < 0.05, **p < 0.01, ***p < 0.001.

### 4.3 Chain mediating effects of learning adaptation and online self-regulated learning

This study examined the hypothesized chain mediating effects of learning adaptability and online self-regulated learning in the relationship between digital literacy and academic achievement. Structural equation modeling (SEM) was employed, controlling for gender, major, and grade level. The significance of indirect effects was evaluated using bootstrapping with 5,000 resamples and 95% confidence intervals.

**Figure 1:**
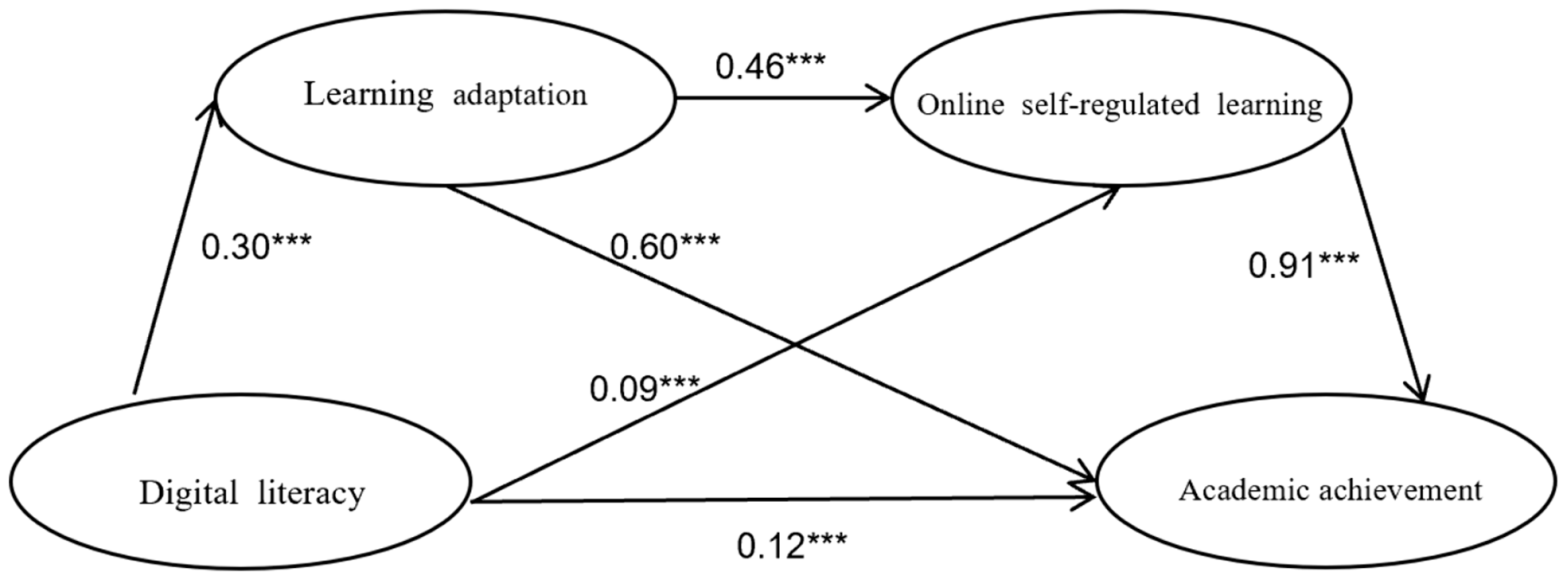
Chain mediation model of learning adaptation and online self-regulated learning between digital literacy and academic achievement.

The proposed structural model, incorporating digital literacy, learning adaptability, online self-regulated learning, and academic achievement, demonstrated a good fit to the data (χ^2^/df = 2.967, SRMR = 0.054, RMSEA = 0.047, CFI = 0.907, TLI = 0.902). As detailed in Figure 1, the standardized path coefficients indicated several significant direct effects. Digital literacy significantly and positively predicted learning adaptability (*β* = 0.30, *p* < 0.001) and online self-regulated learning (*β* = 0.09, *p* < 0 0.001). Learning adaptability, in turn, significantly and positively predicted online self-regulated learning (*β* = 0.46, *p* < 0 0.001) and academic achievement (*β* = 0.60, *p* < 0.001). Furthermore, online self-regulated learning significantly and positively predicted academic achievement (*β* = 0.91, *p* < 0.001). Even with the inclusion of the mediators, the direct path from digital literacy to academic achievement remained significant (*β* = 0.12, *p* < 0.001), suggesting partial mediation.

The results of the bootstrap analysis for specific indirect effects, detailed in Table 2, revealed that digital literacy influenced academic achievement through three significant indirect pathways, as confirmed by 95% confidence intervals that did not contain zero. Firstly, the indirect effect of digital literacy on academic achievement through learning adaptability (digital literacy → learning adaptability → academic achievement) was significant (estimate = 0.161). Secondly, the indirect effect of digital literacy on academic achievement through online self-regulated learning (digital literacy → online self-regulated learning→ academic achievement) was also significant (Estimate = 0.127). Finally, the chain mediation pathway, where digital literacy influenced academic achievement sequentially through learning adaptability and then online self-regulated learning (digital literacy → learning adaptability → online self-regulated learning→ academic achievement), was found to be significant as well (estimate = 0.094). Comparing these magnitudes, the indirect path via learning adaptability alone accounted for the largest proportion (42.15%) of the total indirect effect of digital literacy on academic achievement.

**Table 2:** The chain mediation effect of learning adaptation and online self-regulated learning between digital literacy and academic achievement.

Model pathways	Indirect effect value	Boot SE	Boot LLCI	Boot ULCI	Relative mediating effects
Total indirect effect	0.382	0.025	0.332	0.431	
Indirect effect 1	0.161	0.019	0.126	0.199	42.15%
Indirect effect 2	0.127	0.018	0.093	0.163	33.24%
Indirect effect 3	0.094	0.013	0.069	0.120	24.61%

Boot SE, Boot LLCI, and Boot ULCI refer to the standard error of the indirect effect estimated by the percentile bootstrap method with bias correction, as well as the lower and upper limits of the 95% confidence interval, respectively.

## 5 Discussion

In this study, we examined the impact and mechanisms of digital literacy on academic achievement among college students, focusing on the mediating roles of learning adaptation and online self-regulated learning. The findings provide substantial support for the proposed model, offering a nuanced understanding of these complex relationships within the current educational context, particularly as digital learning environments become increasingly prevalent.

### 5.1 Direct effect of digital literacy on academic achievement

Consistent with Hypothesis 1, the results revealed a significant positive direct effect of digital literacy on college students’ academic achievement. This finding aligns with a growing body of research (e.g., Chaw and Tang, 2024; Ervianti et al., 2023; Park et al., 2021) suggesting that students with higher digital literacy are better equipped to navigate the academic landscape. It is important to contextualize this finding within the specific setting of this study. The pronounced emphasis on academic excellence and the rapid, often government-driven, integration of digital technologies within Chinese higher education may amplify the observed benefits of digital literacy. Specifically, digital literacy encompasses not only the technical skills to use digital tools but also the cognitive abilities to locate, evaluate, synthesize, and communicate information effectively in digital environments (Eshet, 2004; Gilster, 1997). Students who possess these competencies can more efficiently access diverse learning resources, critically assess the credibility of online information, utilize productivity software for assignments, and engage in collaborative learning through digital platforms. Within the Chinese context, where educational attainment is highly valued and often linked to future prospects, the drive to effectively utilize such digital resources for academic pursuits might be particularly strong, potentially influencing the directness and strength of this relationship. These capabilities directly contribute to a more effective learning process and, consequently, better academic outcomes, as they enable students to meet the increasingly digital demands of higher education.

### 5.2 The mediating role of learning adaptation

Furthermore, this study supported Hypothesis 2, indicating that learning adaptation plays a significant mediating role in the relationship between digital literacy and academic achievement. Students with higher digital literacy demonstrated better learning adaptation, which in turn positively influenced their academic success. This pathway suggests that digital literacy empowers students not just to perform tasks, but to effectively adjust to the evolving learning environments and pedagogical approaches prevalent in modern universities. As higher education increasingly incorporates blended learning models and fully online courses (Zhang and Pochuieva, 2023), students’ ability to adapt their learning strategies, manage new technologies, and cope with different instructional methods becomes crucial. Digital literacy facilitates this adaptation by providing students with the confidence and skills to navigate these changes, reduce learning-related stress, and maintain engagement (Wu and Yuan, 2023). When students can successfully adapt to their learning environment, they are more likely to feel competent, motivated, and behaviorally engaged, all of which are strong predictors of academic achievement. Thus, digital literacy fosters an adaptive capacity that translates into improved academic performance.

### 5.3 The mediating role of online self-regulated learning

The findings show that digital literacy enhances students’ capacity for online self-regulated learning, which subsequently leads to better academic outcomes. This is consistent with studies emphasizing the importance of self-regulation in digital learning environments (e.g., Broadbent and Poon, 2015; Barnard et al., 2009). Online self-regulated learning involves a complex interplay of metacognitive, motivational, and behavioral strategies, such as setting appropriate learning goals, managing time effectively, selecting and using relevant learning strategies, monitoring comprehension, and seeking help when needed within an online context. Students with higher digital literacy are better positioned to leverage digital tools and resources to enact these self-regulatory processes. By fostering these online self-regulated learning skills, digital literacy empowers students to take greater control of their learning journey in online settings, leading to more focused effort, deeper understanding, and ultimately enhanced academic achievement.

### 5.4 The chain mediating role of learning adaptation and online self-regulated learning

A noteworthy discovery of this study is a chain mediating effect where digital literacy influences learning adaptation, which in turn affects online self-regulated learning, ultimately impacting academic achievement. This sequential mechanism highlights a more intricate pathway than the simple mediation alone. It suggests that digital literacy first equips students with the foundational skills and confidence to effectively adapt to the demands and characteristics of their (often digital) learning environments (learning adaptation). This enhanced adaptability then creates a more conducive internal and external state for students to successfully engage in online self-regulated learning behaviors. As Dweck and Leggett (1988) suggested, adaptive mindsets can lead to more effective strategy use. Once students feel adapted and competent in the online environment, they are more likely to proactively set goals, monitor their progress, and adjust their online self-regulated learning. This effective self-regulation in the online domain is then the more proximal driver of improved academic achievement.

This chain resonates strongly with Self-Determination Theory (Deci and Ryan, 2012). Digital literacy likely bolsters students’ sense of competence in using digital tools and navigating online spaces. Successful learning adaptation further satisfies the need for competence and fosters a sense of autonomy as students gain mastery over their learning environment. When these fundamental psychological needs for competence and autonomy are met, students are more intrinsically motivated to engage in autonomous, agentic behaviors like online self-regulated learning. This proactive self-management, driven by satisfied needs, logically culminates in improved academic performance, highlighting how digital skills, via adaptation, facilitate the need satisfaction crucial for effective, self-determined learning. Theoretically, this study contributes by integrating digital literacy within an SDT framework and empirically demonstrating a specific sequential mechanism where learning adaptation serves as a crucial bridge, linking digitally-enabled competence to the autonomous self-regulation vital for academic success in contemporary learning environments.

## 6 Implications

This study confirms the significant correlation between digital literacy, learning adaptation, online self-regulated learning, and academic achievement, and it provides a more comprehensive understanding of the relationships among these variables, enriching previous research. In addition to its theoretical value in academia, this study holds significant practical implications for improving college students’ learning adaptation and online self-regulated learning, particularly against the backdrop of the annual increase in college enrolment rates in China, in order to achieve favorable academic performance. We find that digital literacy has become an important factor affecting college students’ academic achievement. College students with higher digital literacy are more likely to adapt to college learning and have more internet self-regulation ability to achieve higher academic achievement.

From the perspective of higher education institutions, it is essential to pay attention to the impact of digital literacy on learning adaptation and online self-regulation in teaching practices. On the one hand, this can be achieved by establishing specialized courses related to digital literacy and actively conducting teaching practice activities to enhance college students’ digital literacy. At the same time, attention should also be paid to the integration of digital literacy in the teaching of professional subjects, introducing digital tools and resources to help students master the use of digital information software and platforms for learning, familiarizing them with learning methods and skills in a digital environment, and cultivating their learning and innovation abilities. On the other hand, teachers’ digital literacy not only affects students’ academic achievements but also has a significant role in promoting students’ digital literacy development, and continuous promotion of teachers’ digital literacy is key to improving students’ digital literacy (Atmojo et al., 2022). Therefore, higher education institutions should also strengthen the training of teachers in digital literacy, enhancing their ability and level to utilize digital technology in teaching, to better guide students in developing digital literacy in their professional studies.

From a more macro perspective, this study also provides references for educational administrative departments regarding higher education reform, instructional design, and talent cultivation. In terms of educational reform, research findings suggest that educational administrative departments should promote higher education institutions to break traditional disciplinary boundaries and construct an interdisciplinary integrated educational ecosystem to respond to the complex and changing social demands of the digital age. For instructional design, educational administrative departments can guide universities to optimize curriculum settings based on research conclusions, systematically embedding digital literacy elements into the curriculum of various majors, rather than simply adding a few elective courses related to digital technology. In terms of talent cultivation objectives, research has provided educational administrative departments with ideas for re-evaluating the positioning of talent cultivation in higher education institutions. It should emphasize the development of students’ lifelong learning abilities and digital adaptability, enabling them to continuously update their knowledge systems in a rapidly evolving digital society. Finally, educational administrative departments can also leverage the findings of this research to enhance their support and supervision of the construction of digital educational resources in higher education institutions, while ensuring the quality and applicability of these digital resources, thereby promoting the entire higher education system to cultivate more talent for society.

In addition, the study also found the chain mediating effect of learning adaptation and online self-regulated learning between digital literacy and academic achievement. This reminds us to emphasize students’ learning adaptability, especially in the current context where digital learning environments are becoming increasingly prevalent. We should help students effectively master learning strategies by offering specialized courses on learning methods, enabling them to quickly adapt to the learning modes of different subjects. In the realm of self-regulated online learning, teachers should guide students to learn how to manage their online learning behaviors autonomously. This can be integrated into daily teaching by incorporating methods for enhancing online learning capabilities and self-monitoring, such as guiding students on how to filter high-quality online learning resources, how to avoid distractions during online learning, and how to use online learning tools for self-assessment and feedback.

## 7 Conclusion

In summary, this study investigated the interplay among digital literacy, learning adaptation, online self-regulated learning, and academic achievement among college students. The findings demonstrate that digital literacy significantly and positively predicts academic achievement. Moreover, this relationship is mediated by both learning adaptation and online self-regulated learning, which operate not only as individual mediators but also form a significant sequential chain from digital literacy, through learning adaptation and online self-regulated learning, to academic achievement.

Although this study has obtained valuable results, some limitations should be acknowledged. Firstly, the sample was drawn from universities within a single province in China, which may restrict the generalizability of the findings to broader regional or national contexts. Future research would benefit from expanding the sampling scope to enhance external validity. Secondly, the findings might be culturally specific. The mechanisms through which digital literacy impacts academic achievement, as well as the mediating roles of learning adaptation and online self-regulated learning, could potentially differ across diverse cultural backgrounds. Therefore, caution is warranted when applying these results internationally, and cross-cultural comparative studies are encouraged. Thirdly, the adoption of a cross-sectional design limits the ability to draw definitive causal inferences and track the developmental nature of academic achievement over time. Future investigations employing longitudinal or experimental designs could provide stronger evidence regarding the observed relationships.

## Data Availability

The raw data supporting the conclusions of this article will be made available by the authors, without undue reservation.

## References

[ref3] Artino A. R. (2008). Cognitive load theory and the role of learner experience: an abbreviated review for educational practitioners. Assoc. Advance. Comput. Educ. J. 16, 425–439. doi: 10.1016/j.ygyno.2015.01.112

[ref4] Atkinson M. Baxter R. Brezany P. Corcho O. (2013). The Data Bonanza: Improving Knowledge Discovery in Science, Engineering, and Business. Wiley-IEEE Computer Society Press.

[ref5] Atmojo I. R. W. Ardiansyah R. Wulandari W. (2022). Classroom teacher's digital literacy level based on instant digital competence assessment (IDCA) perspective. Mimbar Sekolah Dasar 9, 431–445. doi: 10.53400/mimbar-sd.v9i3.51957

[ref6] Azevedo R. Cromley J. G. (2004). Does training on self-regulated learning facilitate students' learning with hypermedia? J. Educ. Psychol. 96:523. doi: 10.1037/0022-0663.96.3.523, PMID: 40526774

[ref7] Barnard L. Lan W. Y. To Y. M. Paton V. O. Lai S. L. (2009). Measuring self-regulation in online and blended learning environments. Internet High. Educ. 12, 1–6. doi: 10.1016/j.iheduc.2008.10.005

[ref9] Boekaerts M. (1999). Self-regulated learning: where we are today. Int. J. Educ. Res. 31, 445–457. doi: 10.1016/S0883-0355(99)00014-2

[ref10] Broadbent J. Poon W. L. (2015). Self-regulated learning strategies & academic achievement in online higher education learning environments: a systematic review. Internet High. Educ. 27, 1–13. doi: 10.1016/j.iheduc.2015.04.007

[ref11] Cazan A. M. (2014). Self-regulated learning and academic achievement in the context of online learning environments. In The international scientific conference Elearning and software for education (3, p. 90). Bucharest: "Carol I" National Defence University.

[ref12] Chaw L. Y. Tang C. M. (2024). Exploring the relationship between digital competence proficiency and student learning performance. Eur. J. Educ. 59:e12593. doi: 10.1111/ejed.12593

[ref13] Cole N. S. (1990). Conceptions of educational achievement. Educ. Res. 19, 2–7. doi: 10.3102/0013189X019003002

[ref14] Corti F. Llanes J. Dorio Alcaraz I. Freixa Niella M. (2023). Initial adaptation among university student: the case of the social sciences. PLoS One 18:e0294440. doi: 10.1371/journal.pone.0294440, PMID: 37956193 PMC10642847

[ref15] Dabbagh N. Kitsantas A. (2012). Personal learning environments, social media, and self-regulated learning: a natural formula for connecting formal and informal learning. Internet High. Educ. 15, 3–8. doi: 10.1016/j.iheduc.2011.06.002

[ref16] Deci E. L. Ryan R. M. (2012). Self-determination theory. Handbook of theories of social psychology, 1, 416–436. SAGE Publications.

[ref17] Dweck C. S. Leggett E. L. (1988). A social-cognitive approach to motivation and personality. Psychol. Rev. 95:256. doi: 10.1037/0033-295X.95.2.256

[ref18] Erstad O. Kjällander S. Järvelä S. (2021). Facing the challenges of ‘digital competence’ a Nordic agenda for curriculum development for the 21st century. Nord. J. Digit. Lit. 16, 77–87. doi: 10.18261/issn.1891-943x-2021-02-04

[ref19] Ervianti E. Sampelolo R. Pratama M. P. (2023). The influence of digital literacy on student learning. J. Educ. Lang. Teach. Sci. 5, 358–365. doi: 10.52208/klasikal.v5i2.878

[ref20] Eshet Y. (2004). Digital literacy: a conceptual framework for survival skills in the digital era. J. Educ. Multimedia Hypermedia 13, 93–106.

[ref21] Eshet-Alkalai Y. Soffer O. (2012). Guest editorial--navigating in the digital era: digital literacy: socio-cultural and educational aspects. Educ. Technol. Soc. 15, 1–2.

[ref22] Feng T.-Y. Su T. H X.-W. Li H. (2006). Development of the college student learning adaptation scale. Acta Psychol. Sin. 5, 762–769.

[ref23] Gilster P. Watson T. (1997). Digital literacy.

[ref24] Hadwin A. F. Järvelä S. Miller M. (2011). Self-regulated, co-regulated, and socially sharedregulation of learning. Handbook Self Regulation Learn Perform 30, 65–84.

[ref25] Hietajärvi L. Maksniemi E. Salmela-Aro K. (2022). Digital engagement and academic functioning. Eur. Psychol. 27, 102–115. doi: 10.1027/1016-9040/a000480

[ref26] Iliichuk L. Vorobets O. (2020). Psychological and pedagogical features of students' adaptation to studying at higher education institutions. J. Vasyl Stef. Precarpathian Natl. Univ. 7, 184–191. doi: 10.15330/jpnu.7.1.184-191

[ref27] Kauffman D. F. (2004). Self-regulated learning in web-based environments: instructional tools designed to facilitate cognitive strategy use, metacognitive processing, and motivational beliefs. J. Educ. Comput. Res. 30, 139–161. doi: 10.2190/AX2D-Y9VM-V7PX-0TAD

[ref29] Lynch R. Dembo M. (2004). The relationship between self-regulation and online learning in a blended learning context. Int. Rev. Res. Open Distrib. Learn. 5, 1–16. doi: 10.19173/irrodl.v5i2.189

[ref30] Lyu Z. Yao H. (2021). A case study of information education and UNESCO’s actions during the COVID-19. In 2021 2nd International Conference on Big Data and Informatization Education (ICBDIE), IEEE (701–704).

[ref31] Martin A. J. Nejad H. G. Colmar S. Liem G. A. D. (2013). Adaptability: how students' responses to uncertainty and novelty predict their academic and non-academic outcomes. J. Educ. Psychol. 105:728. doi: 10.1037/a0032794

[ref32] Martínez-Bravo M. C. Sádaba Chalezquer C. Serrano-Puche J. (2022). Dimensions of digital literacy in the 21st century competency frameworks. Sustain. For. 14:1867. doi: 10.3390/su14031867

[ref34] Nyström A. S. Jackson C. Salminen Karlsson M. (2019). What counts as success? Constructions of achievement in prestigious higher education programmes. Res. Pap. Educ. 34, 465–482. doi: 10.1080/02671522.2018.1452964

[ref35] Pangrazio L. Selwyn N. (2019). ‘Personal data literacies’: a critical literacies approach to enhancing understandings of personal digital data. New Media Soc. 21, 419–437. doi: 10.1177/1461444818799523

[ref36] Park H. Kim H. S. Park H. W. (2021). A scientometric study of digital literacy, ICT literacy, information literacy, and media literacy. J. Data Inf. Sci. 6, 116–138. doi: 10.2478/jdis-2021-0001

[ref37] Pintrich P. R. De Groot E. V. (1990). Motivational and self-regulated learning components of classroom academic performance. J. Educ. Psychol. 82:33. doi: 10.1037/0022-0663.82.1.33

[ref39] Sheng S. Y. (2022) Research on the construction and application of evaluation indicators for digital literacy of college students. Master's degree thesis, Dalian University of Foreign Languages. Available at: https://link.cnki.net/doi/10.26993/d.cnki.gslyc.2022.000246 (Accessed September 12, 2024).

[ref41] Sousa R. D. Karimova B. Gorlov S. (2020). Digitalization as a new direction in education sphere. E3S Web Conf. 159:09014. doi: 10.1051/e3sconf/202015909014

[ref42] Tekesbaeva N. Kultan Y. Ongarbayeva A. Ibraev A. Yerimbetova Z. (2023). Digital technologies as an adaptive learning tool in higher education. In E3S Web of Conferences (403, p. 08023). EDP Sciences.

[ref43] Tondeur J. van Braak J. Ertmer P. A. Ottenbreit-Leftwich A. (2016). Understanding the relationship between teachers’ pedagogical beliefs and technology use in education: a systematic review of qualitative evidence. Educ. Technol. Res. Dev. 65, 555–575. doi: 10.1007/s11423-016-9481-2

[ref44] Vermunt J. D. Verloop N. (1999). Congruence and friction between learning and teaching. Learn. Instr. 9, 257–280. doi: 10.1016/S0959-4752(98)00028-0

[ref45] Wu W. Yuan L. (2023). The relationship between digital literacy and academic performance of college students in blended learning: the mediating effect of learning adaptability. Advanc. Educ. Technol. Psychol. 7, 77–87. doi: 10.23977/aetp.2023.070813

[ref46] York T. T. Gibson C. Rankin S. (2015). Defining and measuring academic success. Pract. Assess. Res. Eval. 20:n5. doi: 10.7275/HZ5X-TX03

[ref47] Zhang K. Pochuieva O. (2023). Matching degree between university students' digital literacy and the current situation of mobile language learning. Int. J. Interact. Mob. Technol. 17:177. doi: 10.3991/ijim.v17i14.41185

[ref48] Zhou H.-L. (2020). Research on the influence of self-regulated learning on college students' academic achievement (Master's Degree Thesis, Tianjin University). Available online at: https://link.cnki.net/doi/10.27356/d.cnki.gtjdu.2020.003904 (Accessed September 20, 2024).

[ref49] Zhou H. Long L.-R. (2004). Statistical testing and control methods for common method bias. Adv. Psychol. Sci. 6, 942–950.

[ref51] Zimmerman B. J. (2002). Becoming a self-regulated learner: an overview. Theory Pract. 41, 64–70. doi: 10.1207/s15430421tip4102_2

